# Oral tranexamic acid use for melasma is not associated with thromboembolism: Findings from a multicenter propensity score–matched electronic health record cohort

**DOI:** 10.1016/j.jdin.2025.09.005

**Published:** 2025-09-30

**Authors:** Tyler Hernandez, Kayla Penny, Nicholas Culotta

**Affiliations:** a Department of Internal Medicine, LSU Health Sciences Center, New Orleans, Louisiana; b Department of Dermatology, LSU Health Sciences Center, New Orleans, Louisiana

**Keywords:** arterial, blood clot, chloasma, deep vein thrombosis (DVT), general dermatology, medical dermatology, melasma, risk analysis, safety, thromboembolic events, thromboembolism, tranexamic acid, TriNetX

*To the Editor:* Oral tranexamic acid (oTXA) is increasingly prescribed in melasma for its pigment-reducing effect.^1^, ^2^, ^3^, ^4^ However, concerns about thromboembolic risk persist. This study retrospectively evaluated thromboembolism risk in melasma patients treated with oTXA using a propensity score–matched cohort from the TriNetX Research Network.

We queried 108 health care organizations from 2005 to 2024 to identify adults diagnosed with melasma (International Classification of Diseases 10: L81.1). The control cohort included melasma patients without tranexamic acid exposure, and the exposed cohort included those prescribed oTXA 650 mg postdiagnosis. Patients with coagulation disorders, systemic hormone/estrogen modulator use, recent or current pregnancy, malignancy, nicotine dependence (cigarettes), or prior venous thromboembolism (VTE) or arterial thromboembolism (ATE) outcomes were excluded. Propensity score matching based on demographics and thrombotic risk factors yielded 682 well-balanced pairs (Table I). Primary outcomes included VTE, ATE, diagnostic testing for thrombosis, and initiation of anticoagulant/thrombolytic therapy within 120 days. Analyses estimated absolute risk and risk ratios with 95% confidence intervals (Supplementary Methods, available via https://data.mendeley.com/datasets/f9fpvc6pmj/1).

**Table I:** Baseline characteristics of included patients before and after propensity score matching

Variable	Cohort	Unmatched			Matched		
		Patients (mean ± SD)	% of cohort	*P*	Patients (mean ± SD)	% of cohort	*P*
				SMD			SMD
Current age	oTXA	684 (50.8 ± 9.3)	100	.135	682 (50.8 ± 9.3)	100	.803
	Control	48,383 (51.5 ± 12.2)	100	.064	682 (50.7 ± 11.0)	100	.014
Age at index	oTXA	684 (47.7 ± 9.1)	100	<.001	682 (47.7 ± 9.1)	100	.888
	Control	48,383 (46.0 ± 12.1)	100	.158	682 (47.6 ± 10.8)	100	.008
Female	oTXA	652	95.3	<.001	650	95.3	.899
	Control	44,086	91.1	.168	649	95.2	.007
Male	oTXA	27	3.9	.003	27	4.0	.891
	Control	3330	6.9	.130	28	4.1	.007
White	oTXA	249	36.4	<.001	249	36.5	.075
	Control	25,044	51.8	.313	281	41.2	.096
African American	oTXA	94	13.7	.096	94	13.8	.488
	Control	5651	11.7	.062	103	15.1	.038
Asian	oTXA	130	19.0	<.001	128	18.8	.780
	Control	3790	7.8	.332	124	18.2	.015
Other race	oTXA	79	11.5	.063	79	11.6	.131
	Control	4574	9.5	.068	62	9.1	.082
Unknown race	oTXA	120	17.5	.952	120	17.6	.187
	Control	8531	17.6	.002	102	15.0	.072
Hispanic	oTXA	131	19.2	.004	131	19.2	.677
	Control	7316	15.1	.107	125	18.3	.023
Not Hispanic	oTXA	473	69.2	<.001	471	69.1	.814
	Control	26,265	54.3	.309	475	69.6	.013
Hypertension	oTXA	124	18.1	.518	124	18.2	.780
	Control	9244	19.1	.025	128	18.7	.095
Type 2 diabetes	oTXA	40	5.8	.147	40	5.9	.908
	Control	3531	7.3	.059	39	5.7	.006
Dyslipidemia	oTXA	163	23.8	.156	163	23.9	.271
	Control	10,443	21.6	.054	146	22.0	.060
CKD	oTXA	12	1.8	.438	12	1.8	.667
	Control	1060	2.2	.031	10	1.5	.023
Atrial fibrillation	oTXA	10	1.5	.082	10	1.5	1.000
	Control	409	0.8	.058	10	1.5	<.001
BMI, kg/m^2^	oTXA	357 (27.1 ± 6.1)	52.2	.058	355 (27.1 ± 6.1)	52.1	.398
	Control	25,739 (27.8 ± 6.6)	53.2	.105	369 (26.7 ± 6.2)	54.1	.063
BMI <18.5	oTXA	23	3.4	.697	23	3.4	.662
	Control	1501	3.1	.015	26	3.8	.024
BMI 18.5-25	oTXA	218	31.9	.040	216	31.7	.419
	Control	13,694	28.3	.078	230	33.7	.044
BMI 25-30	oTXA	184	26.9	.992	184	27.0	.807
	Control	13,024	26.9	<.001	180	26.4	.013
BMI 30-35	oTXA	101	14.8	.231	101	14.8	.054
	Control	7971	16.5	.047	77	11.3	.105
BMI 35-40	oTXA	59	8.6	.726	59	8.7	.124
	Control	3994	8.3	.013	44	6.5	.083
BMI 40-45	oTXA	25	3.7	.759	25	3.7	.656
	Control	1879	3.9	.012	22	3.2	.024
BMI 45-50	oTXA	11	1.6	.633	11	1.6	.826
	Control	898	1.9	.019	10	1.5	.012
BMI ≥50	oTXA	10	1.5	.570	10	1.5	1.000
	Control	591	1.2	.021	10	1.5	<.001

Control = melasma without TXA.

*BMI*, Body mass index; *CKD*, chronic kidney disease; *oTXA*, oral tranexamic acid; *SD*, standard deviation; *SMD*, standardized mean difference.

Over 120 days, the incidence of VTE (1.6%), diagnostic testing for thrombosis (1.8%), and initiation of anticoagulant or thrombolytic therapy (1.6%) were identical between oTXA and control cohorts. The oTXA cohort had no observed ATEs. Extending follow-up to 180 and 365 days had no significant effect on VTE, testing, or treatments; only controls experienced ATE (1.5%; risk ratio not estimable: zero events in oTXA cohort) (Table II).

**Table II:** Outcomes in propensity-matched cohorts

Outcome	Time (d)	oTXA *n*/*N* (%)	Control *n*/*N* (%)	*P* value	RR (95% CI)
Venous thromboembolism	120	10/628 (1.6)	10/634 (1.6)	.983	1.01 (0.42-2.41)
	180	10/628 (1.6)	10/634 (1.6)	.983	1.01 (0.42-2.41)
	365	11/628 (1.8)	10/634 (1.6)	.809	1.11 (0.48-2.60)
Arterial thromboembolism	120	0/680 (0.0)	0/677 (0.0)	–	NE
	180	0/680 (0.0)	10/677 (1.5)	.001	NE
	365	0/680 (0.0)	10/677 (1.5)	.001	NE
Diagnostic testing for thrombosis	120	10/559 (1.8)	10/582 (1.7)	.928	1.04 (0.44-2.48)
	180	13/559 (2.3)	10/582 (1.7)	.466	1.35 (0.60-3.06)
	365	21/559 (3.8)	21/582 (3.6)	.894	1.04 (0.58-1.89)
Anticoagulant or thrombolytic initiation	120	10/632 (1.6)	10/625 (1.6)	.980	0.99 (0.42-2.36)
	180	10/632 (1.6)	10/625 (1.6)	.980	0.99 (0.42-2.36)
	365	10/632 (1.6)	15/625 (2.4)	.299	0.66 (0.30-1.46)

Control = melasma without TXA.

*CI*, Confidence interval; *NE*, not estimable (due to zero events in experimental group); *oTXA*, oral tranexamic acid; *RR*, risk ratio.

Our analyses align with the growing evidence that oTXA use is not associated with increased thromboembolism risk in melasma. A registry study involving approximately 2 million women treated for menorrhagia identified a 4-fold relative increase in VTE risk with a 15 g/5-day oTXA course, yet only one excess VTE per 78,549 treated and no ATE signal despite higher and more frequent dosing than used in melasma.^5^ A 2025 systematic review of 51 randomized controlled trials reported no thromboembolic events with daily oTXA doses of ≤4 g over 900 patient-years of exposure.^4^ Although long-term data are limited, a recent case series monitored 26 women for an average of 17 months and found no thrombotic complications, suggesting prolonged low-dose regimens may be well tolerated.^3^ In a cohort of 561 melasma patients, 90% improved after a median 4-month oTXA course, with only one case of deep vein thrombosis occurring in a patient later diagnosed with hereditary protein S deficiency.^2^ oTXA therapy appears well tolerated across specialties, with rare adverse events typically confined to patients with preexisting risk factors.^1^, ^2^, ^3^, ^4^, ^5^

Limitations include retrospective design and potential for selection bias. Although propensity score matching was employed, residual confounding may persist. Reliance on diagnostic coding may not capture all relevant clinical events. Time-varying changes after cohort entry (eg, smoking initiation) were not captured. Because cohorts necessitated different index event definitions, immortal time bias must be considered. The observational design also limits the ability to draw definitive causal inferences.

The present study reinforces the safety of 650 mg oTXA as an adjunctive therapy in melasma, with no observed increase in short-term thromboembolic risk among appropriately screened patients. Clinician assessment of thromboembolic risk factors remains essential, and additional research is necessary to clarify long-term risks and cumulative dose thresholds.

## Conflicts of interest

None disclosed.
